# Occurrence of microplastics in freshwater gastropods from a tropical river U-Taphao, southern Thailand

**DOI:** 10.7717/peerj.14861

**Published:** 2023-02-08

**Authors:** Preyanuch Jitkaew, Siriporn Pradit, Prakrit Noppradit, Karnda Sengloyluan, Mathinee Yucharoen, Suvit Suwanno, Varaporn Tanrattanakul, Kittiwara Sornplang, Thongchai Nitiratsuwan

**Affiliations:** 1 Coastal Oceanography and Climate Change Research Center, Faculty of Environmental Management, Prince of Songkla University, Hat Yai, Songkhla, Thailand; 2 International College, Prince of Songkla University, Hat Yai, Songkhla, Thailand; 3 Faculty of Environmental Management, Prince of Songkla University, Hat Yai, Songkhla, Thailand; 4 Faculty of Science, Prince of Songkla University, Hat Yai, Songkhla, Thailand; 5 Faculty of Science and Fisheries Technology, Rajamangala University of Technology Srivijaya, Sikao, Trang, Thailand

**Keywords:** Snail, River, Food chain, Biota

## Abstract

**Background:**

Microplastics (MPs) are pollutants in rivers and marine environments. Rivers can be sources and sinks of MPs that enter the biota. Previous studies focusing on freshwater species are quite limited, especially for gastropods. Freshwater gastropods are essential to aquatic ecosystems because they are food to other aquatic animals, such as fish, shrimp, and crabs. They are a crucial link in the food chain between water resources and human food. Therefore, this study aimed to investigate MP accumulation in freshwater gastropods, commonly known as snails (*Filopaludina sumatrensis* *speciosa* and *Pomacea canaliculata*), in a river flowing into a shallow coastal lagoon.

**Method:**

In this study, snail tissue samples were digested with 30% hydrogen peroxide. The mixture was heated at 60 °C for 24 h. MP particles were identified, counted, and characterized (shape, size, and color) by visual identification under a stereomicroscope. Furthermore, polymer-type identification was performed using Fourier transform infrared spectroscopy (FTIR). Analysis of variance (ANOVA) was applied for the statistical analysis.

**Results:**

The MPs found were as follows: 4.76 particles/individual were found in *F. sumatrensis speciosa* upstream, 5.20 particles/individual were found in *F. sumatrensis speciosa* downstream, 7.28 particles/individual were found in *P. canaliculata* upstream, and 4.00 particles/individual were found in *P. canaliculata* downstream. It was found in the two-way ANOVA that the accumulation of MPs in gastropods was affected by species and study sites (upstream and downstream). There was a significant difference in the amount of MPs in *P. canaliculata* between upstream and downstream sites (*p* = 0.003). Fibers were the most common MPs in both species. Moreover, *P. canaliculata* upstream had the most significant amount of MPs. The smallest amount of MPs was recorded for *P. canaliculata* downstream, but there was great diversity in shape, size, and polymer type. MPs sized 500 μm^–1^ mm were the most common in both species. Fourier transform infrared spectroscopy revealed six polymers: poly (ethylene terephthalate), polypropylene, rayon, polyethyleneimine, polyamine, and poly **(**propylene: ethylene). The occurrence of MPs in gastropods is alarming for food security in Thailand. The results of this study can be used to support baseline data on MP accumulation among freshwater gastropods.

## Introduction

Plastic waste in aquatic ecosystems has become a local, regional, and global issue. Several million tons of plastic waste are released into the marine environment through rivers and atmospheric particles (Chen et al., 2021; Pradit et al., 2022a). Microplastics (MPs) are plastic debris defined as particles less than 5 mm (NOAA, 2009) and are classified as marine waste. MPs may harm the environment once they enter the food chain (Goh et al., 2021; Pradit et al., 2022b). Several previous studies on MPs have focused on the marine environment, such as in sediment (Pradit et al., 2022c; Pradit et al., 2021), beach sand (Jualaong et al., 2021; Pradit et al., 2020a; Pradit et al., 2020b), seawater (Prarat & Hongsawat, 2022), or aquatic biota (*e.g*., fish, bivalves, shrimp, jellyfish, and bristle worm) (Azad et al., 2018; Hamid et al., 2018; Lusher, McHugh & Thompson, 2013; Goh et al., 2021).

Mollusks are essential components of the main benthic community and are abundant at the bottom of natural water bodies. Mollusca consists of a group of Gastropoda and Bivalvia classes and is essential to aquatic ecosystems in terms of feeding for other aquatic animals, such as fish, shrimp, and crabs. Shellfish are a crucial link in the food chain of water resources (Mercogliano et al., 2020). Recently, many wastewater and pollutants, including MPs, have entered rivers, making it impossible for shellfish to live and potentially leading to their loss because MPs have direct biological effects by disturbing the behavioral response of freshwater gastropods, hence increasing their vulnerability to predation (Seuront, 2018). MP contamination in gastropods ranged from 0.25 to 764.81 particles/individual (Putri & Patria, 2021; Zaki et al., 2021). The study of MP contamination in biota is vital since it can help assess the situation of MPs in the aquatic food chain.

Additionally, studying MP contamination may indicate potential MP hazards to human health because the toxicity of MPs accumulated in marine biota can be transmitted into the food chain up to humans. The impact of MPs on aquatic biota livelihood is currently under study. For example, MPs increase stress on fish and affect their swimming behavior (Ašmonaitė et al., 2018). However, little research has been conducted on the impacts of MPs on freshwater organisms living in rivers, such as snails. There have been many reports evaluating MPs in river waters (Eo et al., 2019; Jiang et al., 2019; Lestari et al., 2020). Rivers transport between 1.15 and 2.41 million tons of plastic waste to the seas (Siegfried et al., 2017). Because rivers are a significant source of MPs, MPs are likely ingested by biota living in river systems. Rivers are also wastewater sinks from municipalities that release untreated water from industry (Phutmongkhol, Thongyoi & Jungcharoentham, 1999). During the COVID-19 pandemic, MPs were found in fish from Songkhla Lagoon, with the most common type being rayon polymer fibers (Pradit et al., 2021).

Gastropods, namely *Filopaludina sumatrensis speciosa* and *Pomacea canaliculata*, have a scattered distribution worldwide and are most common along rivers, where local people in tropical countries consume them. *P. canaliculata* is an invasive species imported from South America to Southeast Asia and Thailand in 1981 for aquaculture and food consumption (Brito, Carvalho & Joshi, 2016). Therefore, people resort to freshwater gastropods as alternative protein sources (Jiang et al., 2019). Studying MPs in gastropods can indicate MP contamination in freshwater systems. Additionally, it serves as a bioindicator of environmental pollutants because humans are the primary consumers in the food chain, raising awareness of microplastic pollution and food safety (Azhari et al., 2022; Multisanti et al., 2022; Cera et al., 2022; Hu, Shen & Tang, 2022; Courtene-Jones et al., 2022). To date, there have been limited studies on MP contamination in freshwater gastropods living in rivers in tropical areas. Therefore, this study aims to (i) investigate MP contamination in two gastropod species (*F. sumatrensis speciosa* and *P. canaliculata*) and (ii) study the abundance of MPs found in gastropods living upstream and downstream of the river.

## Materials and Methods

### Study area and sample collection

The U-Taphao Canal is the name of the leading natural river flowing to Songkhla Lagoon, which contacts the Gulf of Thailand. From upstream to downstream, the U-Taphao Canal is 130 km long, has a winding path upstream, and is straight when it enters the lower lowlands of the lagoon. The average depth of the U-Taphao Canal is between 4–10 m, and the average canal width is ~40 m long. The U-Taphao Canal is an important water reservoir in the flood defenses of the nearby city of Hat Yai. The upstream section was selected for study because anthropogenic activities, such as fishing and agriculture, were assumed to contribute to MPs. The downstream area includes the agricultural, fishing, industrial, and commercial industries.

**Figure 1:**
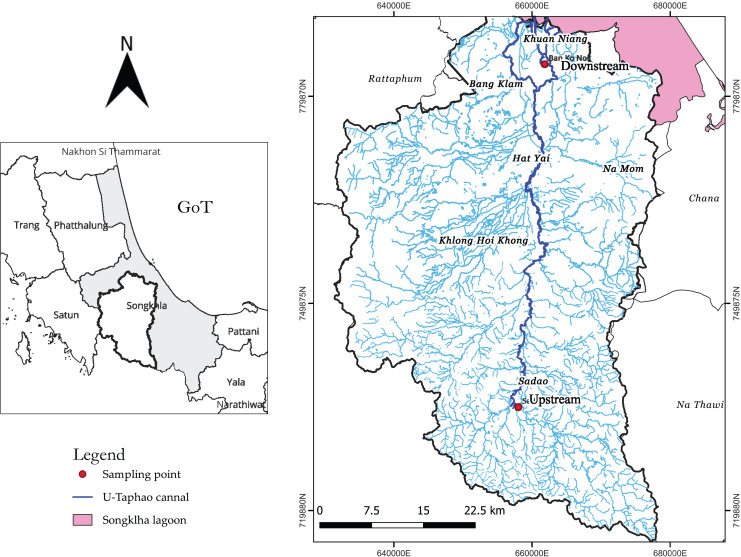
Map showing the sampling stations (upstream and downstream) on the U-Taphao Canal, Songkhla Province, Southern Thailand.

Two gastropod species, *F. sumatrensis speciosa* and *P. canaliculata*, in the U-Taphao Canal, were collected in February 2022, immediately after the villagers manually caught them. The first area was upstream of the river (6°38′57.12″N, 100°25′34.08″E), and the second area was downstream of the river (7°5′41.53″N, 100°27′55.69″E; Fig. 1). The villagers caught the mollusks during the day and put the gastropods in a clean metal bucket. A total of 419 individual gastropods were collected: 68 individuals of *F. sumatrensis speciosa* were caught upstream, 150 individuals were caught downstream, 149 individuals of *P. canaliculata* were caught upstream, and 52 individuals were caught downstream. A researcher transferred all samples to clean and wide glass bottles and preserved them in an icebox (4 °C) to prevent airborne and human contamination. Next, the samples were transported back to the laboratory. The gastropods were stored in a freezer at −20 °C for further analysis.

### Laboratory analysis

#### MP analysis

The preserved gastropod samples were analyzed at Prince of Songkhla University, Thailand. The optimized protocols from the Food and Agriculture Organization of the United Nations (Carpenter & Niem, 1998) were used to assess the size and weight of the whole body of each sample. A random sampling of 100 (25 *F. sumatrensis speciosa* upstream, 25 *P. canaliculata* upstream, 25 *F. sumatrensis speciosa* downstream, and 25 *P. canaliculata* downstream) individual gastropods from the areas mentioned above (*n* = 419) was conducted. MP extraction was performed following the optimization protocol outlined in Yu et al. (2019). The preserved gastropod samples were cleaned using deionized water to remove the wood chips, and the entire flesh was carefully removed from the shell without breaking it apart. Next, the calcareous operculum was removed without any meat sticking to it so that only the wet tissue could be weighed to 0.01 mg accuracy by placing the tissue on a preweighed Petri dish and using a weighing machine Ohaus (Model PA4102; Pioneer, Tokyo, Japan). The tissue sample was then put into a 250 mL beaker. The samples were treated with 50 mL of 30% hydrogen peroxide (H_2_O_2_) following an adapted protocol from Zaki et al. (2021) and Xu et al. (2020).

Additionally, the effectiveness of the digestion protocols for the biogenic organic matter was studied by Pfeiffer & Fischer (2020), who found that the application of H_2_O_2_, showed the highest efficiency of biogenic organic matter digestion in soft and hard tissues. The mixture was heated at 60 °C for 24 h and stirred for 2 min every 2 h. Finally, the beaker was covered with aluminum foil to prevent airborne contamination, and the remaining liquid was filtered with a 20 µm cloth filter to keep only the MPs. The particles were placed in Petri dishes and stored in a 50 °C hot oven to dry for ~4 h.

#### MP identification

MP particles were identified, counted, and characterized (shape, size, and color) by visual identification under a stereomicroscope (Leica, EZ4W; Switzerland, Europe) with the Leica Application Suite program. We also applied the Hidalgo-Ruz et al. (2012) rules mentioned in Pradit et al. (2022a) to assist in identifying the MPs most encountered in this study. The rules were as follows: rule 1 = no cellular or organic structures visible, rule 2 = fibers should be equally thick throughout their entire length, and rule 3 = particles should exhibit homogenous color throughout the item. The morphotypes of MPs were classified into fiber, fragment, and other shapes (modified from Li et al. (2016)). MP sizes were classified into four sizes, *i.e*., <100 µm, 100–500 µm, >500–1 µm, and >1 mm (modified from Karbalaei et al. (2019)). After that, the shape, size, and color of each item were recorded.

Furthermore, polymer-type identification was performed using attenuated total reflection FTIR (ATR-FTIR) (Spectrum Two; Perkin Elmer Spectrum IR version 10.6.2, spotlight 200i, UK). The wavelengths used were 4,000–400 cm^−1^ with a resolution of 4 cm^−1^ and were analyzed with Spectrum IR software using the attenuated total reflection mode. The composition of the plastics was determined by comparing the sample with the spectral libraries.

#### Contamination prevention

Distilled water was filtered before being used in the experiment to avoid MP contamination. A blank test was undertaken to observe the possible airborne contamination of the Petri dishes. The laboratory had no disturbances, such as wind or smoke. Wearing gloves, lab coats, and masks was mandatory during the experiments. Chemical experiments were conducted under a fume hood to prevent dangerous and volatile chemicals from escaping and airborne contamination from the room.

### Data analysis

Statistical analysis was performed using MS Excel (Office Professional Plus 2019; Microsoft Corp., Redmond, WA, USA) and R. All data were tested for normal distribution and variance homogeneity before statistical analysis. The analyses of variances (one-way and two-way ANOVA) were used to determine the interactions between species, study sites, and the amount of MPs accumulated in the gastropods. A t-test was used to compare MP differences between two groups of gastropod species in two areas. Correlation tests were used to study the relationship between size and MPs accumulation within a gastropod, with the significance level set at *p* < 0.05.

## Results

### Gastropod sizes

MPs were found in 100 analyzed gastropod samples from upstream and downstream stations. Gastropod samples **(**whole body with shells**)** were measured for their width, length, and weight before quantified analysis. It was indicated by these results that the average length of apple snails downstream was the largest, followed by *F. sumatrensis speciosa* downstream, *P. canaliculata* upstream, and *F. sumatrensis speciosa* upstream. The average weight of *P. canaliculata* downstream was the highest, followed by *P. canaliculata* upstream, *F. sumatrensis speciosa* upstream, and *F. sumatrensis speciosa* downstream (Table 1).

**Table 1:** Size and weight measurements of gastropods from the U-Taphao Canal, in southern Thailand.

Gastropod	Station	Tissue weight (g)	Shell length (mm)	Shell weight (mm)	Amount of MPs (particles/individual)
*Filopaludina sumatrensis speciosa*	Upstream	1.48 ± 0.65	25.13 ± 3.26	3.32 ± 1.38	4.76 ± 0.56
	Downstream	1.79 ± 0.52	28.08 ± 2.20	3.22 ± 0.83	5.20 ± 0.51
*Pomacea canaliculata*	Upstream	3.36 ± 2.29	33.19 ± 7.92	7.39 ± 4.19	7.28 ± 0.77
	Downstream	5.60 ± 1.77	37.90 ± 4.15	10.0 ± 3.20	4.00 ± 0.52

### MPs found in gastropods

MPs were found in all 100 analyzed gastropod samples, with 533 particles recorded.

**Figure 2:**
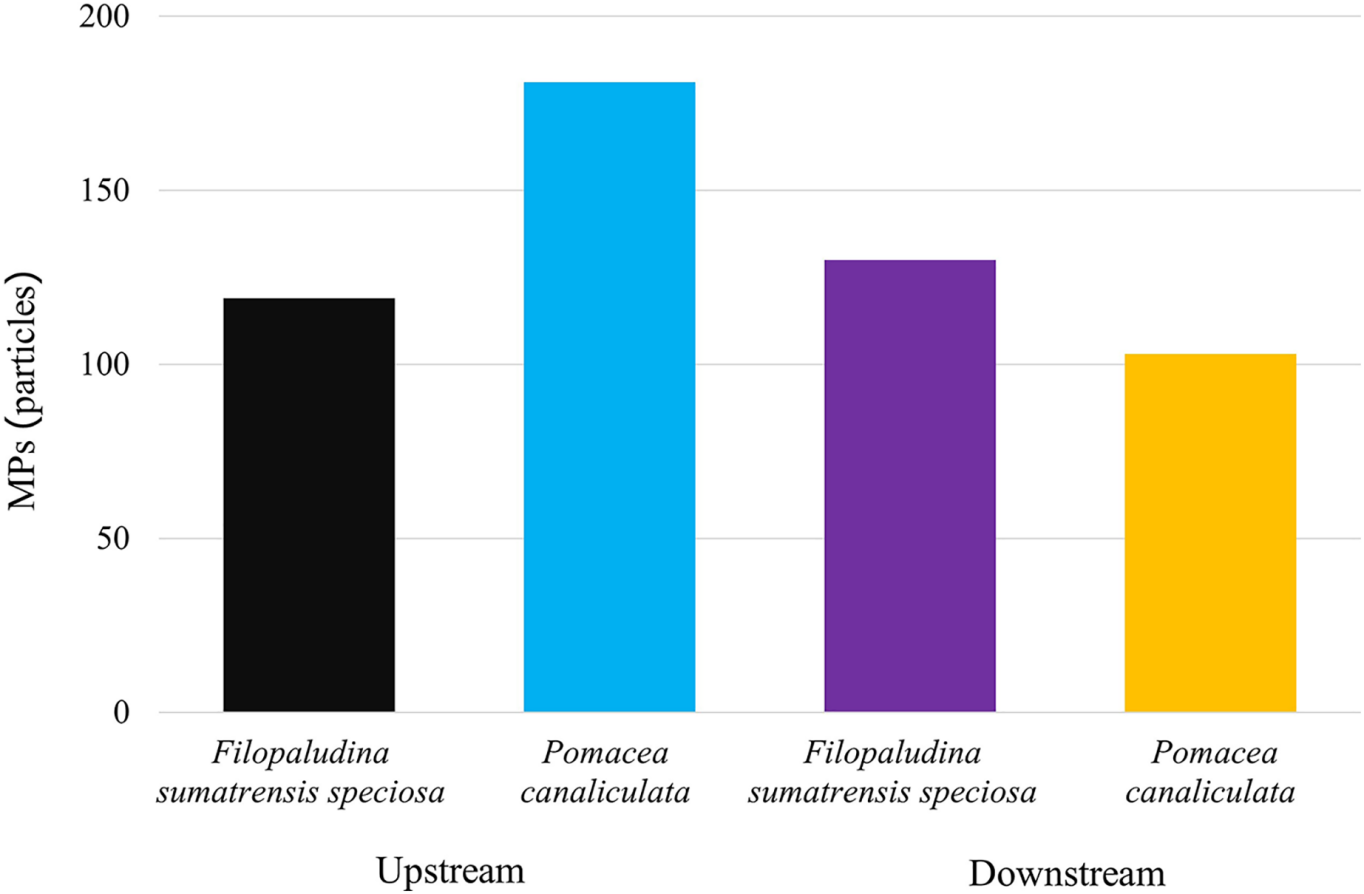
MP abundance in gastropods along the U-Taphao Canal.

Many factors increase MP accumulation in gastropods, such as the area (study site) and the species. It was found using the two-way ANOVA that the accumulation of MPs in gastropods affected species and study sites (upstream and downstream) (*p* = 0.003). There were significant differences in MP abundance between sites according to one-way ANOVA: *F. sumatrensis speciosa* upstream, *F. sumatrensis speciosa* downstream, *P. canaliculata* upstream, and *P. canaliculata* downstream (*p* = 0.002). There was a significant difference in the amount of MPs in *P. canaliculata* between upstream and other sites. From the t-test, the comparison of MP abundance from two stations **(**upstream and downstream**)** with *F. sumatrensis speciosa* showing no significant difference (*p* = 0.564). In contrast, a significant difference was found for *Pomacea canaliculata* (*p* = 0.001). *P. canaliculata* upstream was the most MP-contaminated species (Fig. 2). Comparing the MP abundance for the two species from the upstream station showed no significant difference between *F. sumatrensis speciosa* and *P. canaliculata* (*p* = 0.209). At the same time, *F. sumatrensis speciosa* and *P. canaliculata* from the downstream station also showed no significant difference (*p* = 0.203).

**Figure 3:**
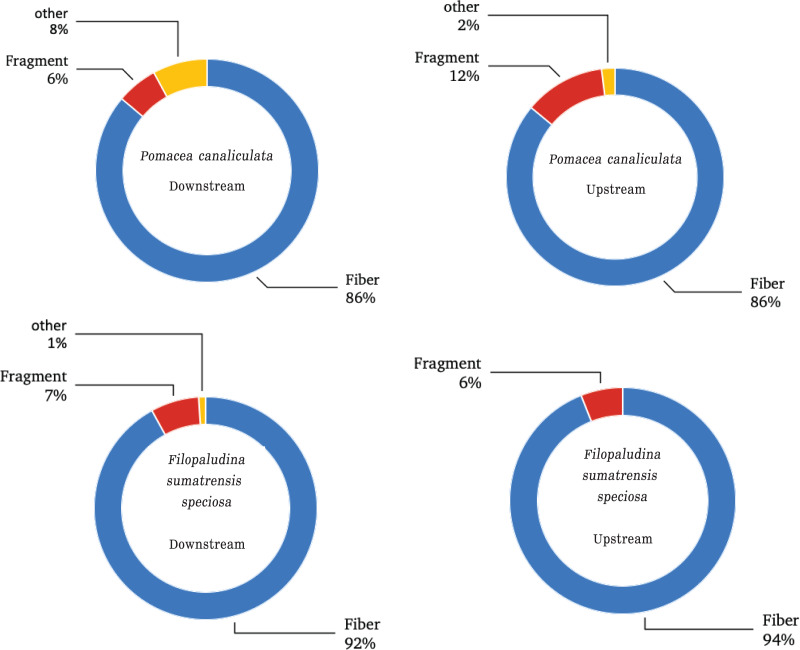
Shapes of MPs in *Filopaludina sumatrensis speciosa *and *Pomacea canaliculata*.

**Figure 4:**
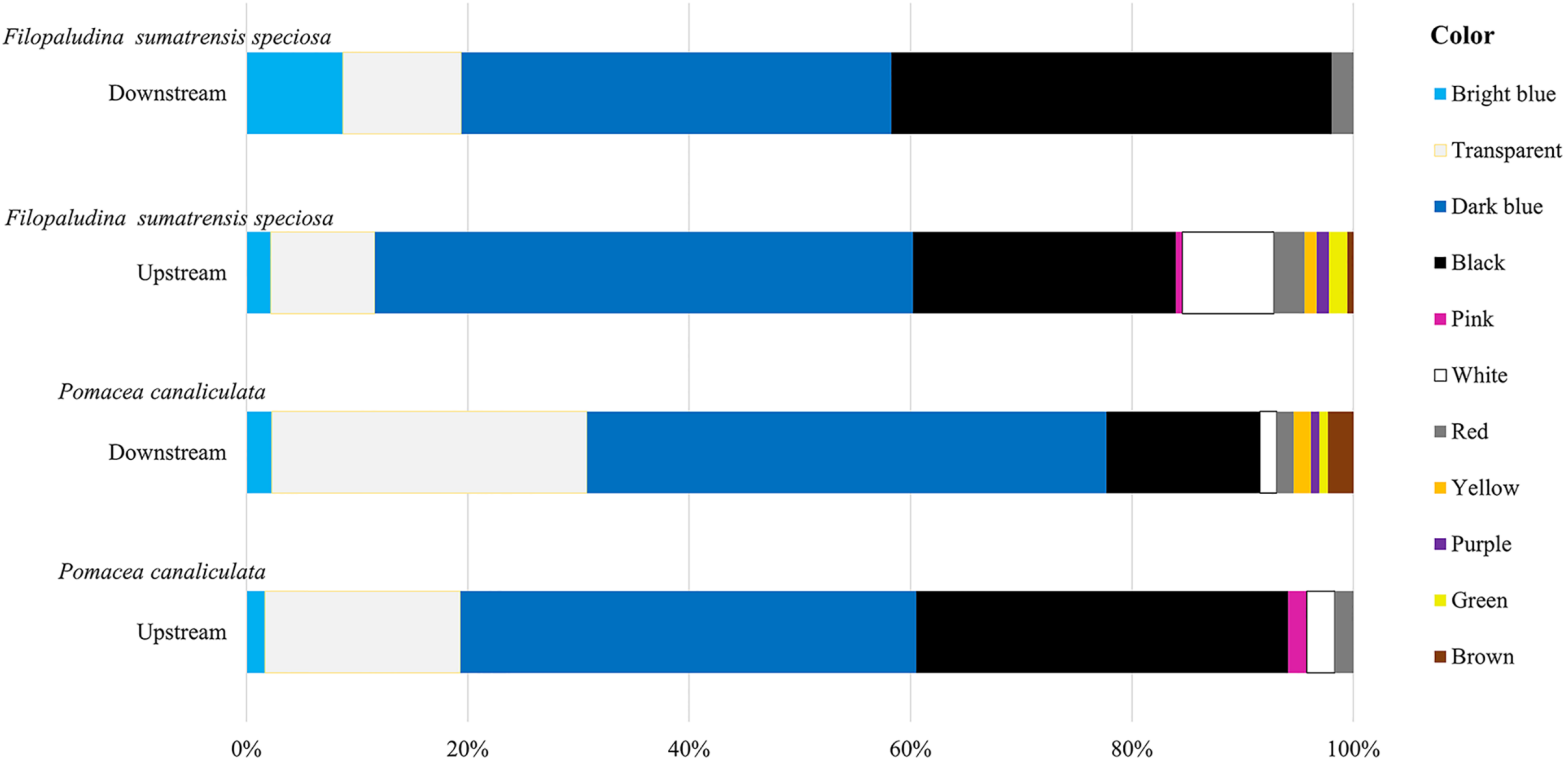
Color of MPs in *Filopaludina sumatrensis speciosa* and *Pomacea canaliculata* upstream and downstream.

**Figure 5:**
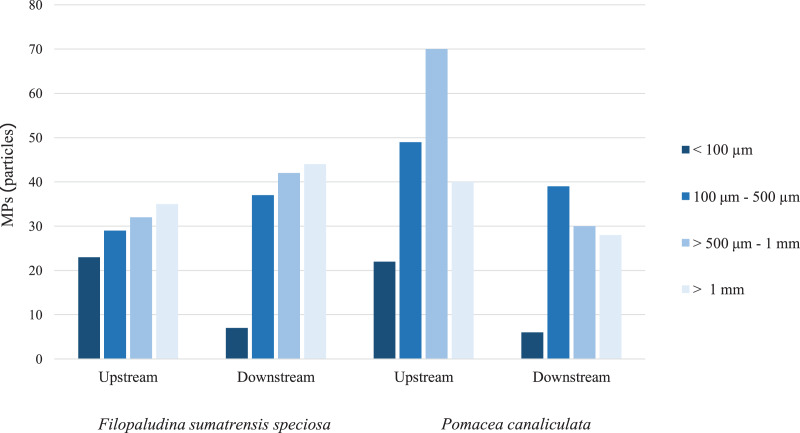
Size of MPs in *Filopaludina sumatrensis speciosa* and *Pomacea canaliculata* upstream and downstream.

Based on all gastropod samples, regarding the shapes of MPs, fibers were the most common shape, with 478 particles (90%), followed by fragments with 38 particles (7%), and other shapes (foam-like round plates and rods) with 17 particles (3%). Fibers and fragments were found in every station, whereas other shapes were observed only in *P. canaliculata* downstream. The largest amount of fibers was recorded in *P. canaliculata* downstream, with 157 particles (29%; Fig. 3). The prevalent colors of MPs were dark blue with 238 particles (45%), black with 142 particles (27%), and transparent with 86 particles (16%). The other colors were white with 20 particles (4%), bright blue with 18 particles (3%), red with 11 particles (2%), and other colors with 18 particles (3%, namely pink, yellow, purple, green, and brown). According to the color characteristics, blue, black, transparent, and red colors were found in the gastropods from every station (Fig. 4). Regarding the size of MPs (Fig. 5), there were four MP sizes in the study, with the predominant sizes being >501 μm^–1^ mm with 174 pieces (33%), 100–500 μm with 154 particles (29%), >1 mm with 147 pieces (28%), and <100 µm with 58 particles (11%). *P. canaliculata* upstream had the most MPs >500 μm^–1^ mm with 70 particles (13%).

### Types of polymer

**Figure 6:**
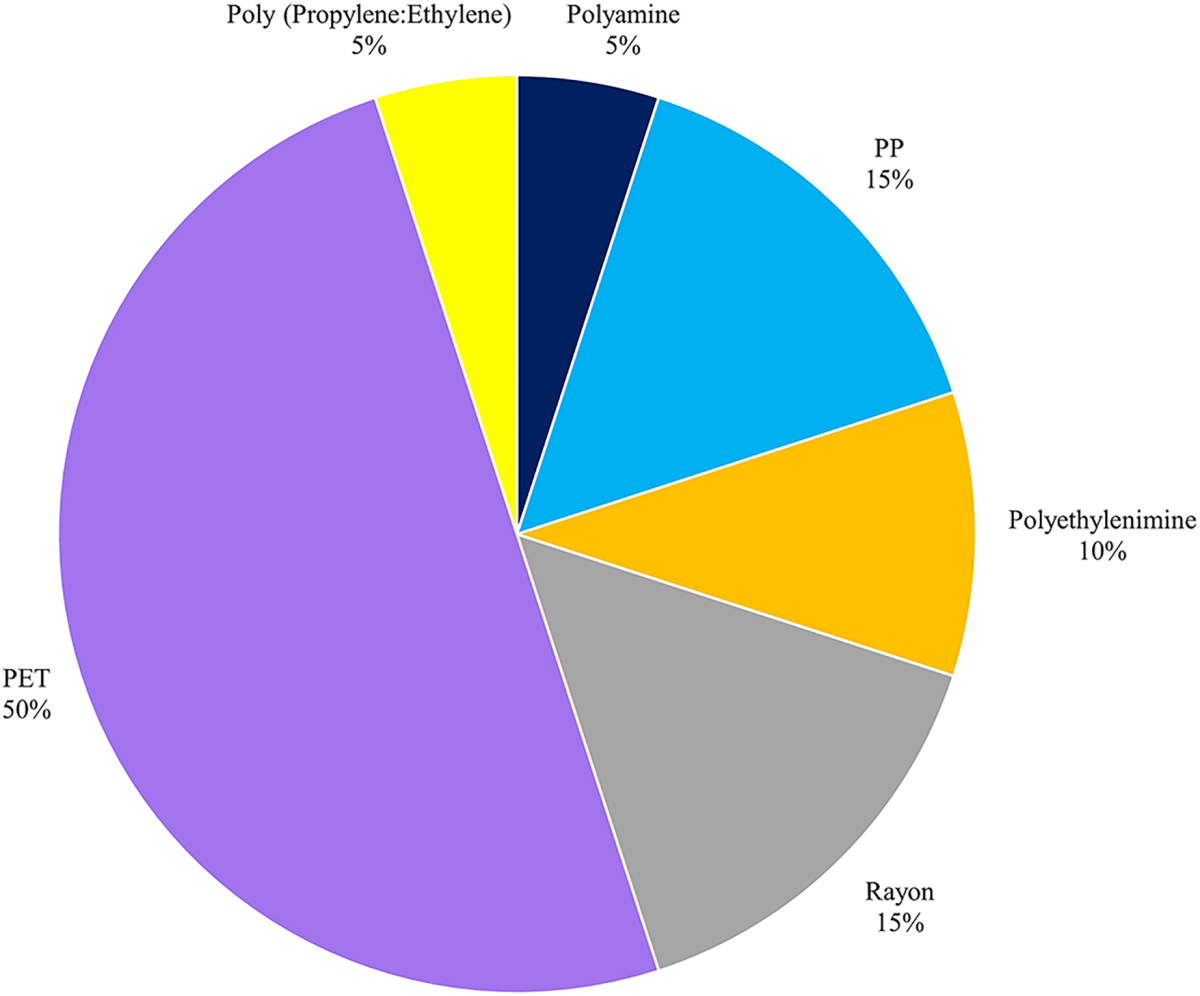
Polymers in gastropods.

**Figure 7:**
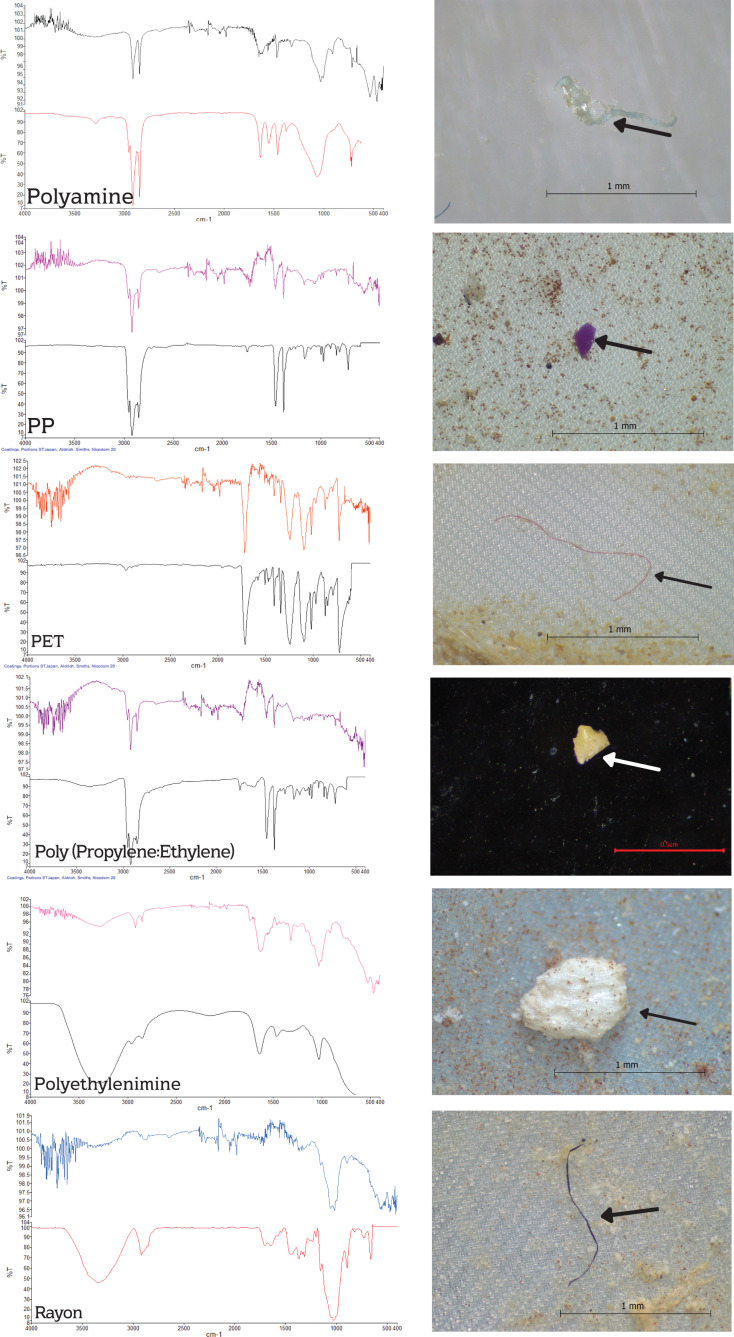
Polymer composition showing the spectrum of MPs of sample gastropods (top line) and spectrum reference (bottom line).

Six polymers were revealed in the FTIR analysis: poly (ethylene terephthalate) (PET) with 50%, polypropylene (PP) with 15%, rayon with 15%, polyethyleneimine with 10%, polyamine with 5%, and poly (propylene: ethylene) with 5**%** (Fig. 6). Polyamine was found only in *P. canaliculata* downstream, whereas PP and polyethyleneimine were found only in *P. canaliculata* from both stations. Copolymer poly (propylene: ethylene) was found only in *F. sumatrensis speciosa* upstream samples, rayon was found only upstream, and PET was found in all gastropod samples. Based on FTIR polymer identification, the spectra of PP and poly (propylene: ethylene) were similar because poly (propylene: ethylene) is a copolymer with a PP substrate (Fig. 7).

### Correlation between the size of gastropods and MP ingestion

**Figure 8:**
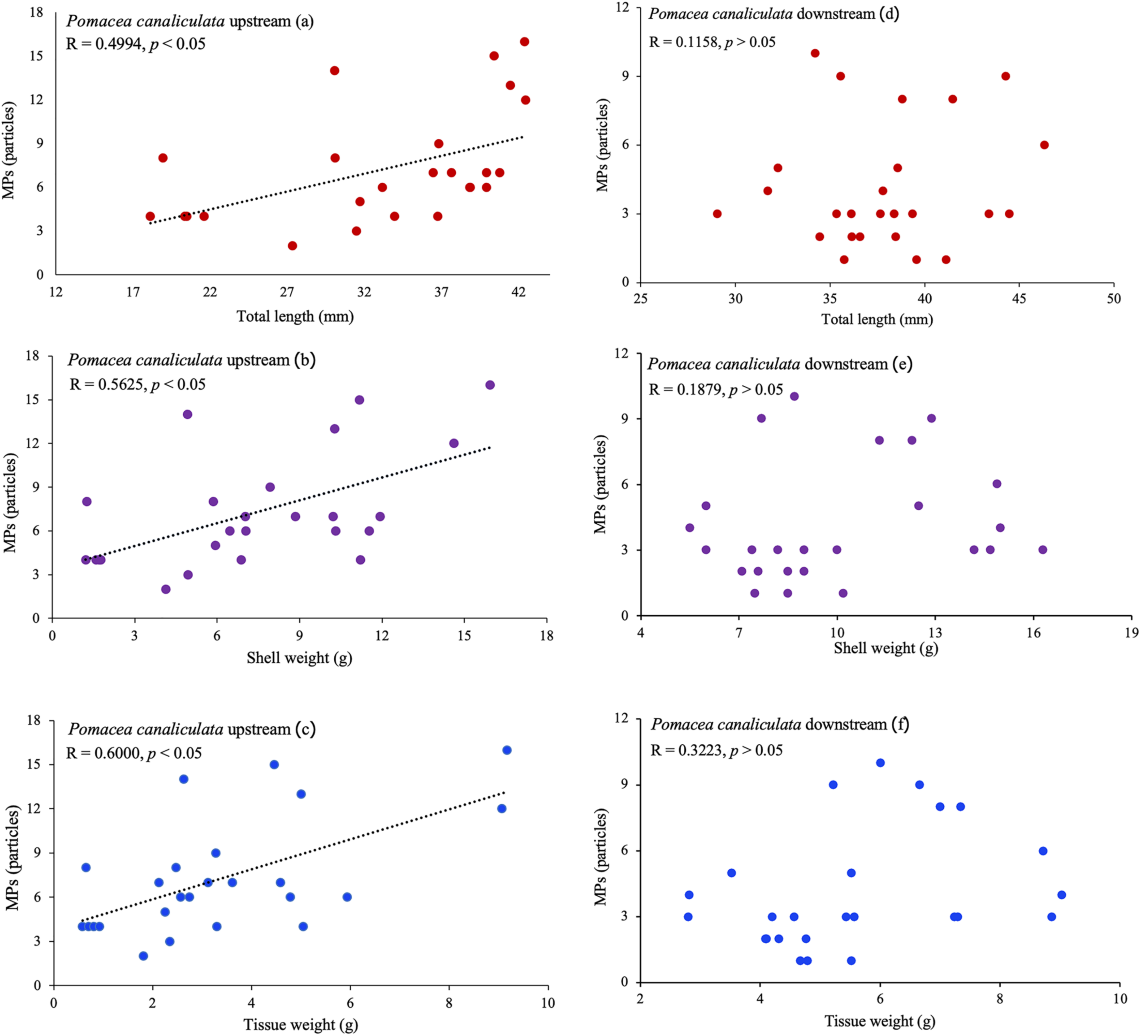
Correlation between the length and weight and the amount of MPs in *Filopaludina sumatrensis speciosa* and *Pomacea canaliculata* (A–F).

*P. canaliculata* upstream showed a positive correlation between the total length, shell weight **(**body weight**)**, and tissue weight of gastropods with MP ingestion (r = −0.4994, r = −0.5625, and r = −0.6000, respectively; Fig. 8). This indicated that the number of MPs ingested by *P. canaliculata* in the upstream site was affected by the size of the mollusks: the larger and heavier the mollusk, the higher the MP ingestion. However, no correlation was observed for either gastropod downstream.

## Discussion

In this study, MPs were found in both gastropod species. The amount of MPs in gastropods depends on the density of MP and the elevation of gastropods where they forage (Kiss, 2017). PET has a density of 1.37–1.45 g/cm^3^, which is heavier than water, making it more likely to float in the middle of the water mass. This is not the case with PP (0.85–0.93 g/cm^3^), making it more likely to float on the water surface (Gilioli et al., 2017; Plastic Europe, 2014; Schwarz et al., 2019). However, there are factors to discuss because they can influence MP ingestion, eating habits, environmental factors, density, and the source of MPs. The feeding habits of *P. canaliculat*a differ from freshwater species. *P. canaliculata* is an alien species with diverse food sources in water and on land (Gilioli et al., 2017; Sun et al., 2012). They can eat various aquatic plants, such as seaweeds, morning glory, water mimosa, *Azolla* spp., water lettuce, chufa, leaf blade, rice sprouts, and even carcasses rotting in the water, depending on their habitat. On average, *P. canaliculata* eats up to 50% of its body weight in food. Food is either ingested directly or kept for later consumption by sending part of the food into an oral cavity filled with sharp teeth (Pradabphetrat et al., 2017). Unlike *F. sumatrensis speciosa*, they use their feet to adhere to materials in water or sink in the mud. They feed by scraping teeth through food attached to materials such as moss, seaweed, water surfaces, plankton, and decaying organic matter. *F. sumatrensis speciosa* has large gills to filter food, with the food combined with a lump of mucus and transported to the head for later consumption (Grairut, 2021).

*P. canaliculata* upstream had more significant MP contamination than downstream specimens, meaning that *P. canaliculata* is highly contaminated with MPs due to prolonged environmental exposure. The amount of MPs was recorded for *P. canaliculata* downstream, but there was a great diversity of shape, size, and polymer type. MPs have several colors that can indicate the characteristics of dyed plastic products, such as clothing, plastic bags, and the color of fishing nets or toys (Thetford, Chorlton & Hardman, 2003). The most common size range of MPs found is 0.5–1 mm, potentially due to the foraging behavior of gastropods, which reflects the distribution of different sizes of MPs.

PET particles were the most frequently found in this study. All polymers found were secondary MPs that probably broke down from clothes, different types of plastic bags, fishing nets, and tendons from fishing rods, similar to Sun et al. (2012). During the COVID-19 pandemic, MPs were found in fish from the Songkhla Lagoon, with rayon being the most common type (Pradit et al., 2021). Polymers in gastropods from other studies included PE-PDM, polyester, polyurethane poly(vinyl acetate), PET, PS, and polyamides (Doyle et al., 2020; Pradabphetrat et al., 2017; Xu et al., 2020; Zaki et al., 2021). This represents various MP contaminations derived from human activities that can be transmitted into the food chain through gastropod accumulation in the canal. Based on this study, 4.76 ± 0.52–5.20 ± 0.51 particles in *Filopaludina sumatrensis speciosa* and 4.00 ± 0.52–7.28 ± 0.77 particles in *P. canaliculata* were found in individuals.

Freshwater gastropods are the most contaminated with fiber MPs compared to MPs contaminating gastropods worldwide (Table 2). MP contamination occurred in gastropods in various countries: 0.68 ± 0.96 particles/individual were found in Ireland found (Doyle et al., 2020), 0.92 ± 1.21 particles/individual were found in Italy (Panebianco et al., 2019), 0.25–0.88 particle/individual were found in Malaysia (Zaki et al., 2021), and 6.1 ± 2.0 particles/individual were found in China (Xu et al., 2020). Our MP results were similar to the studies of Azhari et al. (2022) and Xu et al. (2020) and were lower than the reports of Yasaka et al. (2022), Putri & Patria (2021); Patria, Santoso & Tsabita (2020); Li et al. (2020), and Akindele, Ehlers & Koop (2019). The abundance found depends on the gastropod species in the research, the features of the research areas, the season, and the fate of plastic products on land and in water, which can differ. From this study, most particles found were fibers, similar to the findings reported by other researchers (Kärkkäinen & Sillanpää, 2021; Patria, Santoso & Tsabita, 2020; Doyle et al., 2020; Zaki et al., 2021) since fibers of dissociated plastics can stay on the water surface for a long time due to their low density (Lie et al., 2018). Fishing nets usually degrade, resulting in fiber waste. This is also the case with synthetic fibers from clothes washing in homes or urban communities (Kärkkäinen & Sillanpää, 2021; Montarsolo et al., 2018; Napper & Thompson, 2016).

**Table 2:** MP pollution in gastropod samples worldwide.

Gastropod	Sampling area	Shape	MP abundance	Ref.
*Nerita articulata* (*n* = 67 individuals) *Nerita polita* (*n* = 14 individuals) *Chicoreus capucinus* (*n* = 14 individuals)	Malaysia	Fibers and Fragment	0.25–0.88 particle/individual 0.50–1.75 particles/g	Zaki et al. (2021)
*Telescopium* (Linnaeus, 1758)	Indonesia	–	764.81 particles/individual	Putri & Patria (2021)
*Bellamya aeruginosa*	China	Fiber and fragment	6.1 ± 2.0 items/individual	Xu et al. (2020)
*Lottia scabra* (10 samples)	Indonesia	Fiber, film, and fragment	75.5 particles/individual	Patria, Santoso & Tsabita (2020)
*Littorina littorea* (135 individuals)	Ireland	Fiber and fragment	0.68 ± 0.96 MPs/individual	Doyle et al. (2020)
*Helix aspersa* (44 samples), *Helix pomatia* (4 samples), and *Helix aperta* (37 samples)	Italy	Fragment, foam, film, line and pellet	0.92±1.21 particles/sample	Panebianco et al. (2019)
*Ellobium chinense*	China	–	7 ± 2 – 53 ± 6 items/kg	Li et al. (2020)
*Lanistes varicus* (*n* = 10)	Nigeria	Fiber and film	17 MPs/individual	Akindele, Ehlers & Koop (2019)
*Melanoides tuberculata* (*n* = 10) and *Theodoxus fluviatilis* (*n* = 10)		Fiber	17, 7 MPs/individual	
*Nerita articulata*	Indonesia	Film, fiber, fragment, pellet	3.25–5.75 particles/individual	Azhari et al. (2022)
*Filopaludina martensi* (*n* = 24)	Thailand	Fragment	26.33 ± 33.30 pieces/snail	Yasaka et al. (2022)
*Pomacea canaliculate* (*n* = 24)			11.50 ± 16.80 pieces/snail	
*F. sumatrensis speciosa* and *P. canaliculata*	Thailand	Fiber and fragment	4.96 ± 2.67 particles/individual	This study
			5.64 ± 3.67 particles/individual	

## Conclusions

This study is the first investigation of MPs in freshwater gastropods (*F. sumatrensis speciosa* and *P. canaliculata*) in the study area. Freshwater gastropods (*F. sumatrensis speciosa* and *P. canaliculata*) from the U-Taphao Canal were sold for human consumption and were found to be contaminated with MPs. The most common type of MP found was fibers. The great diversity in the MPs recorded for *P. canaliculata* in terms of shape, size, and polymer type was shown by the results. This implies that invasive species tend to ingest more varieties of MPs than indigenous *F. sumatrensis speciosa*. Information relating to MPs found in dominant gastropods that live in the river flowing into the lagoon is crucial for baseline data. Therefore, regular monitoring of MPs in river water, gastropods, and other aquatic organisms should be a concern. Understanding the mechanisms underlying the interaction of MPs in the biota and MPs in the river environment should be a priority in the future. Subsequently, further research should focus on the concentration of MPs in river water and sediment. Moreover, some challenges remain in assessing human health risks related to gastropod consumption.

## Supplemental Information

10.7717/peerj.14861/supp-1 Supplemental Information 1 Raw data measurement of gastropod from the U-Taphao, Thailand.

10.7717/peerj.14861/supp-2 Supplemental Information 2 Raw data: Polymer Type.

## Additional Information and Declarations

### Data Availability

The following information was supplied regarding data availability: The raw measurements are available in the Supplemental Files.
